# The Effects of Natural Iridoids and Anthocyanins on Selected Parameters of Liver and Cardiovascular System Functions

**DOI:** 10.1155/2020/2735790

**Published:** 2020-03-31

**Authors:** Maciej Danielewski, Agnieszka Matuszewska, Beata Nowak, Alicja Z. Kucharska, Tomasz Sozański

**Affiliations:** ^1^Department of Pharmacology, Wroclaw Medical University, Jana Mikulicza-Radeckiego 2, 50-345 Wroclaw, Poland; ^2^Department of Fruit, Vegetable, and Plant Nutraceutical Technology, Wroclaw University of Environmental and Life Sciences, Chelmonskiego 37, 51-630 Wroclaw, Poland

## Abstract

The old adage says, “you are what you eat.” And although it is a banality repeated by many with a grain of salt, it also has quite a bit of truth in it, as the products we eat have a considerable impact on our health. Unfortunately, humanity is eating worse from one year to another, both in terms of product quality and eating habits. At the same time, it is brought up frequently that plant products should form the basis of our diet. This issue was also reflected in the new version of the food pyramid. Iridoids and anthocyanins are groups of plant compounds with proven beneficial effects on health. Both groups affect the cardiovascular system and the liver functions. Although many mechanisms of action and the therapeutic effects of these compounds have already been learned, intensive animal and clinical research is still underway to explore their new curative mechanisms and effects or to broaden our knowledge of those previously described. In this article, we review the effects of natural iridoids and anthocyanins on selected parameters of liver and cardiovascular system functions.

## 1. Introduction

In the era of fast life and the constant pursuit of wealth, fame, and realization of various dreams, the time spent on preparing meals and caring for a proper, balanced diet has been reduced to a minimum by many people. This situation is being met by food producers, frequently large global food concerns, whose main motto, understandably from an economic point of view, is often to obtain only the highest possible financial profit. Highly processed products, containing many different chemical additives, e.g., flavor enhancers or preservatives; ready meals, which you only need to heat in a microwave; and finally fast food or sweetened drinks, are an increasing part of people's diet. This leads to global health and social consequences, which without exaggeration can be called an epidemic of unhealthy nutrition. Worldwide, diseases such as type II diabetes, atherosclerosis, hypertension, metabolic syndrome, and balance obesity are constantly observed.

Fortunately, opinions calling for a change of lifestyle, including paying more attention to the fact that plant products—fresh and low processed—should be the basis of diet, are becoming louder, including in the mass media. This belief has been confirmed by numerous, recent publications that have meta-analyzed previous studies and have shown significant correlations between increased consumption of fruits and vegetables and decreased risk of coronary artery disease (CAD), cardiovascular disease (CVD) mortality, and stroke [1, 2]. Similarly, a plant-based diet was reported to demonstrate a hepatoprotective capacity in nonalcoholic fatty liver disease (NAFLD) or alcoholic cirrhosis and to contribute to a reduction in cholesterol, alanine aminotransferase (ALT), and aspartate aminotransferase (AST) levels and an improvement in detoxifying processes [3–7].

The best example of a change in the global approach to nutrition is the fact that WHO has recently modified the multiyear guidelines for creating a healthy eating pyramid. In its new version, it is plant products that have a far dominant role. It seems that the simplest advice for those looking for a way to improve their eating habits is to eat more plants. There are many groups of plant compounds showing a confirmed beneficial effect on human health. Such groups are, e.g., iridoids and anthocyanins. These compounds have been known for many years, but their new, potentially valuable therapeutic and prophylactic properties are still being discovered. The aim of this article is to review the reports on the effects of natural iridoids and anthocyanins on selected parameters of liver and cardiovascular system functions.

## 2. Iridoids

**Figure 1:**
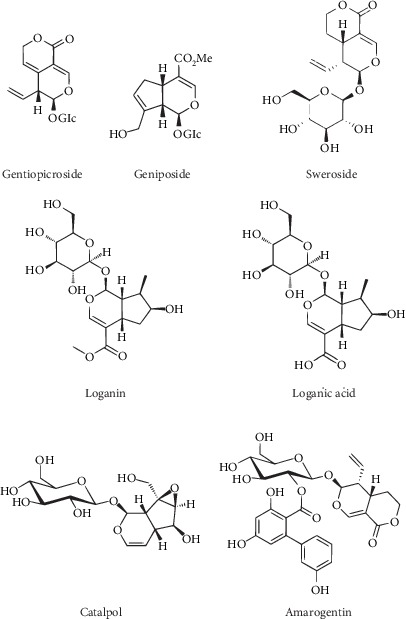
Chemical structures of common iridoids.

Iridoids are a group of organic chemical compounds from the monoterpenoid group. They are found in many plant families, e.g., *Apocynaceae*, *Gentianaceae*, *Lamiaceae*, *Loganiaceae*, *Rubiaceae*, *Scrophulariaceae*, and *Verbenaceae* [8], usually as glycosides with a glucose moiety attached to C-1 in the pyrene ring. Large amounts of iridoids are also observed in herbs with bitter effects. Structurally, they are cyclopentano-(c)-pyran monoterpenoids, and biogenetically and chemotaxonomically, they determine a structural link between terpenes and alkaloids [9]. The basic structural feature of iridoids is a bicyclic H-5/H-9*β*, *β*-*cis*-fused cyclopentanopyran ring system; also, several enantiomeric forms of iridoids exist in nature, e.g., secoiridoids in which the cyclopentane ring is torn between C-7 and C-8 [10]. Iridoids are found in the green parts of plants, mainly in the leaves and young stems, and sometimes in fruits and sprouts. Among the most commonly mentioned iridoids in the therapeutic context, the following stand out above all: gentiopicroside, geniposide, sweroside, loganin, loganic acid, catalpol, and amarogentin (Figure 1) [8, 11–25].

### 2.1. Iridoid Mechanisms of Action and Potential Curative Effects

**Figure 2:**
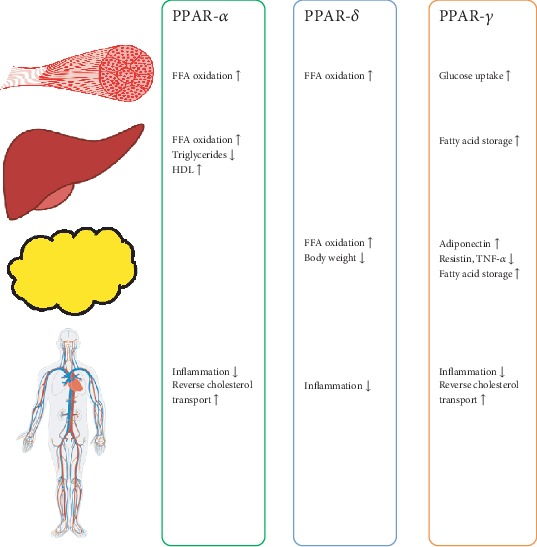
Influence of PPARs on muscles, liver, adipose tissue, and vessel walls.

**Figure 3:**
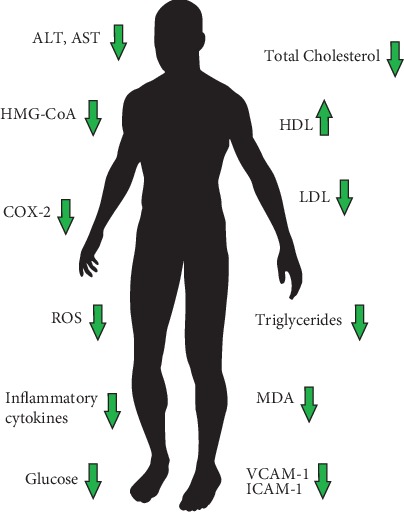
Confirmed and proposed effects and parameters altered by iridoids in humans.

During numerous studies on the biological activity of iridoids, a very wide range of action on the human body was found. Those confirmed so far include cardiovascular, hypoglycemic, hypolipidemic, antihepatotoxic, choleretic, anti-inflammatory, antispasmodic, antitumor, antiviral, immunomodulatory, and purgative activities [11]. The health-improving properties of iridoids are majorly due to their antioxidative and anti-inflammatory activity. The anti-inflammatory effects of iridoids are attributed to the inhibition of cyclooxygenase 2 (COX-2), proinflammatory cytokines, prostaglandin E2 (PGE2), and thromboxane-B2 (TBX2) [12]. Some iridoids, like the hydrolyzed derivative of harpagide, have a similar chemical structure to PGE2 and celecoxib—a selective COX-2 inhibitor [12]. Previous studies have also shown that iridoids may increase the expression of transcription factors involved in the regulation of lipid metabolism, like peroxisome proliferator-activated receptors (PPARs) (Figure 2) and sterol regulatory element-binding proteins (SREBPs) [26, 27]. Among different iridoids possessing anti-inflammatory properties, particularly interesting, due to its possible therapeutic potential with high efficiency of COX inhibition, is loganic acid. Loganic acid has a greater affinity for inducible COX-2 than constitutive COX-1 [28], which means that its therapeutic use does not have to entail severe side effects, such as the risk of gastrointestinal ulceration. In addition to the aforementioned high affinity for COX-2, it reduces the expression of some proinflammatory cytokines, i.a., TNF-*α*, interleukin-1*β* (IL-1*β*), and interleukin-6 (Il-6). It also exerts an inhibitory effect on fMLP-induced superoxide generation in human neutrophils [29]. Confirmed and proposed effects and parameters altered by iridoids in humans are summarized in Figure 3.

### 2.2. Iridoids in Liver Injury

**Figure 4:**
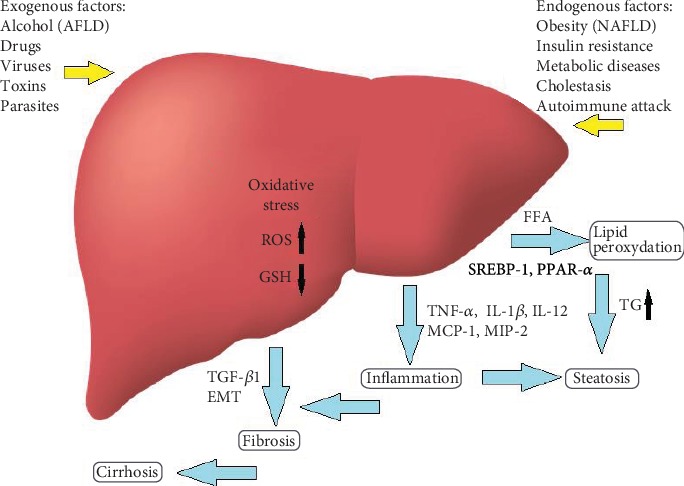
Oxidative stress in the liver, risk factors, follow-up compounds, and mechanisms that are the point of curative action of iridoids and anthocyanins.

The liver is a key organ responsible for the metabolism, excretion, and detoxification processes of the body. Due to its role in the body, it is exposed to injuries from various types of metabolism products, toxins, endotoxins, viruses, or drugs [30]. It is estimated that even about 1,000 commonly used drugs are hepatotoxic. Many cases of liver diseases have been noted every year in the world, and they are also one of the most common causes of death [31]. Oxidative stress is considered the major mechanism contributing to the appearance and progression of liver diseases. The excess of reactive oxygen or nitrogen forms, as well as the deficiency of antioxidant compounds, is the main factor responsible for liver damage [32]. Highly reactive metabolites lead to the depletion of reduced glutathione (GSH) which is needed for the detoxification reaction of glutathione-S-transferase, and also lead to a rise in the levels of lipid peroxides in the liver. This induces, through the nuclear factor-kappa B (NF-*κ*B), the production of proinflammatory cytokines and chemokines, and the activation of cyclooxygenase 2 and inducible nitric oxide synthase (iNOS). Inflammation in the liver is most often mediated by tumor necrosis factor-alpha (TNF-*α*), interleukin-12 (IL-12), monocyte-1 chemotactic protein (MCP-1), and macrophage-2 inflammatory protein (MIP-2) (Figure 4). An increase in TNF-*α* leads to apoptosis and cell death [33, 34].

The hepatoprotective effect of iridoids is mainly attributed to their antioxidant activity. This action is both indirect, through the stimulation of the antioxidant defense system, and direct, through the removal of reactive oxygen species (ROS) [6]. Therefore, the use of plant substances with antioxidant properties, as ready-made preparations or intermediates to obtain drugs with hepatoprotective effects, seems to be justified and desirable. There are many such substances, e.g., silymarin, curcumin, ellagic acid, or exactly iridoids. An example of a mixture of the latter is Picroliv. Picroliv is a standardized mixture of iridoid glycosides isolated from the roots and rhizomes of the *Picrorhiza kurroa*. It contains at least 60% of a mixture of picroside I and kutkoside in a 1 : 1.5 ratio; the remainder (40%) is a mixture of iridoid and cucurbitacin glycosides [35]. Picroliv has antioxidant properties which appear to be mediated through the activity of superoxide dismutase, metal ion chelators, and xanthine oxidase inhibitors [36]. Moreover, Picroliv restores malondialdehyde (MDA) levels to normal in a carbon tetrachloride- (CCl4-) induced liver injury model in mice, thereby pointing to antilipid peroxidative properties. It works on the principle of an anaerobic free radical scavenger, limiting lipid peroxidation involved in cell membrane damage caused by hepatotoxins [34, 35].

Tan et al. conducted an interesting study assessing the hepatoprotective ability of iridoids isolated from *Veronica ciliata* in an acetaminophen-induced acute liver injury murine model. Acetaminophen is one of the most popular analgesic and antipyretic drugs globally, commonly given to adults and children. It turned out that treatment with an isolated iridoid fraction significantly reduced the level of liver enzymes alanine aminotransferase, aspartate aminotransferase, and tumor necrosis factor-alpha in the serum. Decreased malondialdehyde formation and expression of proinflammatory factors, along with elevated superoxide dismutase and glutathione activity in the liver, was also found [37].

One of the basic factors of liver damage, regardless of the cause, is its fibrosis as a result of the deposition of type I collagen in the extracellular space of the liver. Transforming growth factor-*β*1 (TGF-*β*1) plays a key role in the pathogenesis of liver fibrosis (Figure 4). Another important factor is epithelial-mesenchymal transition (EMT) which accompanies the pathological processes and leads to the transformation of the cellular phenotype, including changes in the activity of collagens (I-V) and elastin [13]. Following iridoid compounds, geniposide (present, i.a., in the fruit of *Gardenia jasminoides*) may inhibit TGF-*β*1-induced EMT in hepatic fibrosis by suppressing the TGF-*β*/Smad and ERK-mitogen-activated protein kinase (MAPK) signaling pathways [14]. Catalpol is another iridoid that protects the liver against fibrosis as well as steatosis and necrosis. Catalpol has been reported to reduce serum alkaline phosphatase (ALP), serum ALT, AST, and bilirubin, as well as the liver/body weight ratio. In addition, catalpol limited the fibrosis process by reducing collagen deposition in the liver as well as reduced inflammation by lowering the levels of proinflammatory IL-1*β*, TNF-*α*, IL-18, IL-6, and COX-2 in a dose-dependent manner *in vivo* [15].

In 2018, Dai et al. published the results of a study in which they examined the effect of iridoids, secoiridoids, and their glycosides on acontine-induced hepatotoxicity. The effect of 53 active substances on the human CYP3A4 enzyme was tested. Researchers particularly focused on six popular iridoid compounds: gentiopicroside, sweroside, swertiamarin, loganic acid, 6-O-*β*-d-glucosyl-gentiopicroside, and amarogentin. In particular, the latter showed a significant inductive effect on CYP3A4 mRNA levels in HepG2 cells, as well as in the case of acontine-induced toxicity [16]. These results are consistent with the previously published work of other researchers who confirmed the effectiveness of amarogentin on carbon tetrachloride-induced liver fibrosis in mice. They showed that amarogentin delayed the formation of liver fibrosis and decreased ALT, AST, MDA, and hydroxyproline levels. At the same time, it increased the levels of albumin, cyclic guanosine monophosphate (cGMP), glutathione peroxidase, and superoxide dismutase. In addition, a decrease in the level of *α*-smooth muscle actin, TGF-*β*1, and protein kinases has been observed [17]. Based on the above results, a conclusion that the hepatoprotective effects of iridoids are caused by the facilitation of drug metabolism, amelioration of mitochondrial dysfunction, reduction of oxidative stress, and suppression of the mitogen-activated protein kinase signaling pathway can be drawn.

### 2.3. Iridoids in Fatty Liver Disease

Fatty liver disease is one of the first stages of alcoholic liver damage. Importantly, it is usually reversible. Consumption of large amounts of alcohol contributes to the increase of fatty acid production and impairment of their beta-oxidation in mitochondria, which consequently leads to lipid accumulation in the liver and inflammatory changes within hepatocytes. Sterol regulatory element-binding proteins, primarily SREBP-1c, as well as the PPAR-alpha transcriptional factors, play a major role in controlling fatty acid synthesis and their oxidation. In turn, AMP-activated protein kinase (AMPK), a key factor in controlling cellular energy homeostasis, affects hepatic lipid metabolism through modulating the downstream acetyl-CoA carboxylase (ACC) and carnitine palmitoyltransferase-1 (CPT-1) pathway [18]. Kupffer cells (hepatic resident macrophages) play a pivotal role in the pathogenesis of these inflammatory changes. They express a range of chemokines and proinflammatory cytokines, including IL-1*β* [19]. The release of IL-1*β* requires caspase-1 activation by NOD-like receptors (NLRs), mainly NLRP3, which forms an inflammatory complex. Activation of Kupffer cells leads to the upregulation of inflammatory mediators through toll-like receptor 4 (TLR4). Additionally, too high uric acid or ATP level also plays an important role in the development of inflammation in the course of alcoholic fatty liver disease (AFLD) [20]. High ATP concentration activates the P2X7 receptor, an ATP-gated ion channel, which is considered to be a secondary signal important in the pathogenesis of liver steatosis [21].

Gentiopicroside is an effective and safe therapeutic option in the case of alcoholic fatty liver disease. Li et al. reported that gentiopicroside administered in both acute and chronic alcohol-induced liver impairments in mice reduces the level of serum liver enzymes, as well as the accumulation of triglycerides (TGs). Moreover, it modulates the expression of SREBP-1, PPAR-*α*, and phosphorylated acetyl-CoA carboxylase. Researchers also observed P2X7 receptor suppression and reduction of IL-1*β* production [21]. In turn, geniposide can rebalance a wide range of metabolic disorders due to alcohol-induced liver injury. Zhang et al. identified 48 AFLD-specific biomarkers. Geniposide regulated up to 32 of them. It modified abnormal liver metabolism resulting in the regulation of amino acid metabolism disorders and lessening oxidative stress [22].

The previously mentioned balance obesity associated with improper diet and deficiency of physical activity can lead to nonalcoholic fatty liver disease (NAFLD). Among the factors that may affect the development of NAFLD are, e.g., insulin resistance, oxidative stress, and the action of adipokines (e.g., adiponectin), cytokines, and other inflammatory mediators. Insulin resistance contributes to an increase in lipolysis and concentration of free fatty acids in hepatocytes. This phenomenon together with emerging oxidative stress and mitochondrial dysfunction results in the development of inflammation in the liver and its steatosis [23]. Moreover, overexpression of uncoupling protein-2 (UCP2) in the liver can cause acute liver injury. Genipin (an aglycone derived from geniposide) inhibits UCP2-mediated pyroptosis and reverses liver damage caused by a high-fat diet [24]. Genipin also protects against ischemia and reperfusion- (IR-) induced hepatic injury via improving mitochondrial quality control. It ameliorates hepatocellular oxidative damage and mitochondrial dysfunction triggered by IR [25].

### 2.4. Iridoids in Cholestasis

Liver damage and failure are also commonly caused by cholestasis. Analysis of proteomic and metabonomic studies showed altered pathways in cholestasis-induced liver injury involving increased activity of farnesoid X receptor (FXR)/retinoid X receptor (RXR), bile acid biosynthesis, and peroxisome proliferator-activated receptor-*α*/retinoid X receptor-*α*. Gentiopicroside normalizes metabolic, protein, and blood biochemical markers, as well as alleviates liver damage, so it can be a useful therapeutic alternative in the treatment of cholestasis [38]. It was shown that gentiopicroside modulates bile acid metabolism, which results in a decrease of the intracellular bile acid pool back to basal levels. It upregulates hepatic mRNA levels of synthesis enzymes, transporters, and also ileal bile acid circulation mediators. This effect leads to a decrease in serum and hepatic bile acid levels and a further increase in urinary and fecal bile acid levels [39]. Similar findings were established with the use of another iridoid glycoside—sweroside [40]. It is worth remembering, however, that the hepatoprotective effect of most iridoids is dose-dependent and high doses may cause harmful effects. It has been proven that geniposide at a dose of 300 mg/kg can induce liver injury with accompanying changes in bile acid regulating genes, leading to an accumulation of taurine conjugates in the rat liver [41].

### 2.5. Iridoids in Hyperlipidemia

Hyperlipidemia is a set of metabolic disorders manifested by elevated total cholesterol (often including elevated LDL-C and decreased HDL-C) and triglyceride serum levels. It is most frequently caused by improper nutrition or lack of physical activity or due to genetic predispositions. It evokes excessive fat accumulation in peripheral tissues, especially in arterial blood vessels. It is a direct risk factor for atherosclerosis, cardiovascular diseases, hypertension, diabetes mellitus, nonalcoholic fatty liver disease, dyslipidemia, osteoarthritis, and cancer [42, 43]. Among others, microRNAs (miRNAs) have been reported to play a crucial role in regulating lipid and lipoprotein metabolism. MicroRNAs are short, noncoding, single-stranded RNA molecules which regulate the expression of target genes by partial sequence-specific base-pairing to the targeted mRNA 3′UTR, blocking its translation and promoting its degradation or its sequestration into processing bodies. In the course of hyperlipidemia, increased miRNA levels in circulation are observed [44]. For example, miR-122, through targeting SREBP-1c, regulates fatty acid metabolism, and upregulation of miR-34a results in the downregulation of hepatic PPAR-*α*. Zhong et al. revealed that genipin decreases body weight, lipid serum levels, and hepatic lipid accumulation in high-fat diet mice. It increases the expression of miR-142a-5p, which bounds to SREBP-1c and thus leads to the inhibition of lipogenesis [45]. Another extensive analysis of the curative impact of the iridoid fraction was carried out by Zhu et al. Rats fed a high-fat diet received three different doses of iridoid fraction isolated from *Valeriana jatamansi*. Regardless of the dose, a decrease in weight gain was observed in rats compared to the model group, as well as a decrease in triglyceride and an increase in HDL-C serum levels. In addition, all doses enhanced the expression of apolipoprotein A5 (ApoA5) and the PPAR-*α* receptor and reduced the expression of the SREBP-1c protein in the liver. Moreover, depending on the dose, a different influence of the iridoid fraction was observed, among others, on AST and ALT levels, lipoprotein lipase (LPL) and hepatic lipase (HL) activities, or liver X receptor-*α* (LXR-*α*) expression. These results suggest the positive effect of iridoids on the functioning of the liver and circulatory system in the course of hyperlipidemia. It also indicates the possibility of differentiating some of the desired therapeutic effects depending on the used dose of these substances [46]. Iridoids have also been found to reduce the expression of vascular cell adhesion molecule 1 (VCAM-1) and intercellular adhesion molecule 1 (ICAM-1) as well as the MCP-1 protein, thereby contributing to the reduction of inflammation within the blood vessels. Iridoid glycosides increase the expression of PPAR-alpha and PPAR-gamma, which act as modulators of both acute and chronic inflammation. They also play a key role in regulating energy metabolism, and their agonist may be used in the treatment of dyslipidemia and CVDs [29].

### 2.6. Iridoids in Cardiovascular Disease

One of the most important factors causing cardiovascular disease is type 2 diabetes. At the same time, CVDs are a common cause of mortality in patients with diabetes, mainly due to macrovascular complications such as atherosclerosis. Platelets and shear stress play a major pathophysiological role in the development of atherosclerosis. Atherosclerosis is also a condition directly associated with hyperlipidemia and inflammation in blood vessels. Natural iridoids seem to possess properties that can prevent and reduce the severity of CVDs. *Cornus mas* L. (cornelian cherry) fruits are an interesting, rich source of iridoids, as well as flavonoids including anthocyanins. Sozański et al. conducted a series of studies examining the influence of cornelian cherry on selected parameters of liver and circulatory system functions in various animal models. They proved that administering cornelian cherry significantly reduces serum triglyceride and LDL-cholesterol and increases HDL-cholesterol levels. Moreover, it decreases intima thickness and the intima/media ratio in the thoracic aorta. Cornelian cherry compounds have a substantial protective effect on oxidative stress in the liver and causes a vital enhancement of PPAR-alpha and PPAR-gamma expression in the liver [47–50]. In turn, clinical trials in diabetic type 2 and hyperlipidemic patients showed a significant improvement of the sugar level and insulin secretion in diabetic patients and an amelioration of the lipid profile, apolipoprotein status, and vascular inflammation in hyperlipidemic patients. It was also proven that *Cornus mas* fruits are safe on acute toxicity studies in rat and human models [7].

Catalpol is another iridoid compound the administration of which resulted in significant attenuation of atherosclerotic lesions. It is the main active ingredient of *Rehmannia glutinosa*, *Katalpa officinalis*, or *Euphrasia rostkoviana*. As in the examples quoted earlier, catalpol lowers the levels of total cholesterol, triglycerides, and low-density lipoproteins in blood serum and boosts the level of high-density lipoproteins. Furthermore, the reduction of TNF-*α*, Il-6, MCP-1, soluble VCAM-1, and soluble ICAM-1 levels in the serum was observed in a rabbit study [51]. Meanwhile, the depletion of VCAM-1, MCP-1, TNF-*α*, iNOS, matrix metalloproteinase-9, and NF-*κ*B expression levels in the aortic arch was noticed. The lessening of the lipid peroxidation levels and the enhancement of the antioxidant capacity were also established during the catalpol treatment [51, 52]. Catalpol may be a particularly valuable therapeutic agent in diabetes therapy. It has been proven that it reduces fasting blood glucose (FBG) and random blood glucose (RBG) in a dose-dependent manner, with the reduction of RBG being even greater than in the case of the basic antidiabetic drug—metformin. Moreover, catalpol significantly improves glucose tolerance via increasing insulin sensitivity. The administration of catalpol to the db/db mice significantly increases the expression of 287 genes involved mainly in lipid metabolism, response to stress, energy metabolism, and cellular processes and significantly decreases the expression of 520 genes involved in cell growth and death, in the immune system, and in the response to stress [53]. Catalpol ameliorates hepatic insulin resistance in type 2 diabetes through acting on the AMPK/NOX4/PI3K/AKT pathway [54]. It exerts a renal protective effect in diabetic db/db mice [55] and restores balance between oxidative and antioxidative enzymes in the course of diabetes [56].

One of the suggested mechanisms of the antiatherosclerotic action of iridoid compounds is the inhibition of the 3-hydroxy-3-methyl-glutaryl-CoA (HMG-CoA) reductase. It is a liver enzyme located in the cytoplasm of hepatocytes, responsible for regulating the amount of synthesized cholesterol and thus also affecting its plasma level. The HMG-CoA-inhibiting properties of iridoids, as well as their capacity for lowering the level of total cholesterol, triglycerides, low-density lipoproteins, and very low-density lipoproteins in serum, have been confirmed, i.a., in swertiamarin [57]. Moreover, swertiamarin presents similar signaling pathways as serotonin 5-HT2 receptor modulators. A peripheral increase in serotonin levels is considered to be one of the markers of diabetes. Changing the level of this autacoid implies alterations in the expression of 5-HT receptors which are involved in the pathogenesis of diabetes [58].

Guo et al. reported that geniposide modulates ATP production and glucose-stimulated insulin secretion (GSIS) and increases GLUT2 protein levels in high-glucose concentration in rat INS-1 pancreatic *β* cells [59]. It also reverses glucose-induced impairment of insulin release, induces phosphorylation of AMPK, and inhibits hepatic glucose production. It appears that AMPK plays a key role in geniposide-regulated GSIS in pancreatic *β* cells and glucose production in HepG2 cells [60]. These results are consistent with outcomes of other studies [61, 62]. Moreover, geniposide protects rat insulinoma cells from apoptosis in high-glucose concentrations [63] and regulates endoplasmic reticulum (ER) stress, which might be an important factor of dysfunction and death of pancreatic *β* cells [64]. The antioxidative properties of geniposide eventuates from either the inhibition of numerous pathological processes (e.g., production of some inflammatory cytokines, LPS-induction of nitric oxide, and blocking upstream of TLR4, NF-*κ*B, and MAPK pathways) or the activation of various proteins associated with cell survival, e.g., heme oxygenase-1 (HO-1) and B-cell lymphoma-2 (Bcl-2) or a combination of both. It was proven that geniposide may be useful in the prophylaxis or treatment of not only diabetes mellitus but also cardiac fibrosis, cardiac hypertrophy, myocardium I/R, obesity-related cardiac injury, atherosclerosis, ischemic stroke, and diabetic nephropathy [65].

## 3. Anthocyanins

**Figure 5:**
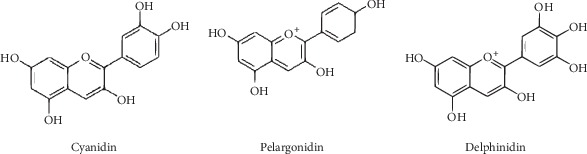
Chemical structures of common anthocyanidins.

Besides iridoids, the other group of natural substances that have garnered increasing interest in recent years are anthocyanins. Anthocyanins are polyphenolic glycosidic plant dyes (mainly red, blue, or purple) of primarily fruits and flowers, which are biochemically related to flavonoids. Structurally, anthocyanins are aliphatic or aromatic three-ring compounds with one or more sugar molecules and sometimes with a sugar-attached aryl group. The colored aglycons are the anthocyanidins (usually cyanidin, pelargonidin, or delphinidin) (Figure 5).

### 3.1. Anthocyanin Mechanisms of Action and Potential Curative Effects

As in the case of iridoids, the overall health-improving properties of anthocyanins seem to result significantly from its antioxidative and anti-inflammatory activity. What distinguishes anthocyanins from other polyphenol compounds is that they are unique in decreasing ROS generation without inducing mitochondrial biogenesis or manganese superoxide dismutase expression [66]. To date, the broad effect of anthocyanins on the liver and cardiovascular function parameters has been reported. The intake of especially anthocyanidins was associated with a statistically significant reduction of cardiovascular disease risk [67, 68]. Anthocyanin's ability to reduce insulin resistance, improve glycemic control, decrease lipid accumulation in the liver, constrain inflammation (manifested as a lessening of inflammatory markers such as TNF-*α*), decrease oxidative stress in the liver (including stress markers such as MDA), and decrease levels of liver enzymes (such as ALT and AST) was also confirmed [69–71].

**Figure 6:**
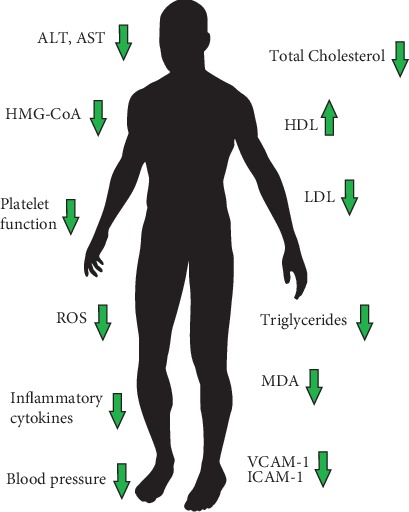
Confirmed and proposed effects and parameters altered by anthocyanins in humans.

The following mechanisms of anthocyanin activity have been proposed, i.a., reduction of cholesterol synthesis through the downregulation of HMG-CoA reductase expression; inhibition of cholesteryl ester transfer protein (CEPT) which lowers the concentration of LDL; reduction of apolipoprotein B and apolipoprotein C-III levels which results in decreasing the concentration of TG in serum; and lessening the levels of various inflammatory cytokines such as IL-6 and IL-1*β*, TNF-*α*, iNOS, and NF-*κ*B in HepG2 cells [70–72]. Anthocyanins were also reported to slow ageing-related deterioration of liver function and structure by inhibiting DNA damage [73]. As anthocyanins potentially inhibit platelet function (e.g., decrease the number of activated platelets and their aggregation), they may represent a useful adjunct in patients treated with typical antiplatelet drugs and be exploited as a complementary therapy to reduce CVD in diabetes [71, 74]. The modulation of the redox state and inflammation by anthocyanins is mediated through various pathways. One of them is the upregulation of the nuclear factor erythroid 2-related factor 2 (Nrf2) [75]. This inducible transcriptional factor is a key regulator of antioxidant responses and contributes to inflammatory tissue injuries. Nrf2 is also responsible for the downregulation of endothelial monocyte chemoattractant protein-1, which is one of the pivotal chemokines regulating the migration and infiltration of monocytes and macrophages [76]. Other proposed mechanisms include the ability to capture free radicals and anions; inhibition of xanthine oxidase—a superoxide-producing enzyme; chelating metal ions, which leads to the formation of stable anthocyanin-metal complexes; and the inhibition of the oxidation of LDL-cholesterol, as well as the modulation of cyclooxygenases and lipooxygenases—the key enzymes in the synthesis of prostaglandins and leukotrienes [71]. Moreover, anthocyanins may protect the heart from ischemia/reperfusion-induced injury by activating signal transduction pathways and sustaining mitochondrial functions [77]. Confirmed and proposed effects and parameters altered by anthocyanins in humans are summarized in Figure 6.

### 3.2. Anthocyanins in Liver Injury

Anthocyanins, like iridoids, may prove effective in the treatment of liver fibrosis. They reduced ALT and AST levels in blood serum; MDA and protein carbonyl content of liver homogenate; and MCP-1, IL-1*β*, MIP-2, collagen III, and *α*-SMA in a mice study [78]. Similar results were obtained in a rat liver fibrosis study [79].

One of the most common anthocyanidin compounds is cyanidin. It occurs in the fruits of hawthorn, hibiscus, elderberry, or blueberry. It exhibits potent antiatherogenic activity *in vitro* and *in vivo* by changing the expression and binding to all three PPAR subtypes, mostly to PPAR-*α*[80]. It was proven that cyanidin-3-O-*β*-glucoside (C3G) possesses the ability of enhancing cellular AMPK activity and ACC phosphorylation and stimulation of carnitine palmitoyltransferase-1 CPT-1 expression, which leads to a significant increase of fatty acid oxidation in HepG2 cells [18]. Similar results were obtained in other studies on anthocyanins [81, 82]. Thereto, C3G lowered fasting glucose levels and improved the insulin sensitivity in both the high-fat diet-fed mice and the db/db mice model. Furthermore, depletion of white adipose tissue messenger RNA levels of MCP-1, TNF-*α*, and IL-6; serum concentrations of TNF-*α*, IL-6, and MCP-1; macrophage infiltration in adipose tissue; and attenuation of liver steatosis were observed. It has been reported that C3G attenuated oxidative stress by activating the GSH synthesis antioxidant defense mechanism against excessive intracellular ROS production, contributing to the prevention of hyperglycemia-induced hepatic oxidative damage [83]. C3G was also shown to regulate the thermogenic and secretory functions of brown adipose tissue (BAT) [84].

### 3.3. Anthocyanins in Cardiovascular Disease

Although there has been quite a lot of research on anthocyanins, their mechanisms of action are not fully known and it has not been thoroughly determined which compounds and in what doses are the most beneficial; therefore, it is worth noting any reports that broaden our knowledge on this issue. Habanova et al. have shown that the regular intake of bilberries (*Vaccinium myrtillus* L.), fruits very rich in anthocyanins, both in women and men causes decreasing LDL-C/TG and increasing HDL-C levels and thus may contribute to reducing the risk of CVDs [85]. Cassidy et al. conducted a series of clinical trials examining the influence of higher intake of fruit-based anthocyanins and flavonoids on some cardiovascular diseases. They are potentially useful in the prevention of hypertension, in the reduction of myocardial infarction (MI) risk in men and young women, and in the reduction of ischemic stroke risk in men. Researchers suggested that a key component underlying the reduction in that risk may be an anti-inflammatory effect of anthocyanins and flavonoids [86–89].

However, it should be taken into consideration that low plasma concentrations and rapid clearance kinetics of anthocyanins suggest that it is their phenolic metabolites which are responsible for their biological activity *in vivo*. The most common theory of anthocyanin metabolism leans on the assumption that their degradation is a result of their chemical instability and the impact of bacterial catabolism, resulting in a number of circulating phenolic metabolites. Warner et al. investigated the influence of physiologically relevant anthocyanin metabolite signatures, derived from C3G, on soluble VCAM-1 and IL-6 in human endothelial cells. It turned out that signatures of anthocyanin metabolites, identified postconsumption of dietary achievable levels of anthocyanins, had inhibitory effects on inflammatory protein secretion, with maximal effects observed for the 6 and 24 h profiles. Researchers also noticed that the greatest inhibition of VCAM-1 occurred in response to the 24 h metabolite signature, which may suggest that the metabolites of lower intestinal microbial origin are responsible for fasting or chronic anti-inflammatory impact [90]. In another study, it was shown that anthocyanin metabolites modify vascular reactivity by inducing HO-1 and modulating NADPH oxidase (NOX) activity in the endothelium, which resulted in decreased superoxide production and ameliorated NO bioavailability [91]. These results were confirmed in a subsequent study, which additionally proved that the bioactivity of common phenolic metabolites of anthocyanins is increased when in combination, indicating their additive or synergistic effects [92].

## 4. Concomitant Intake of Iridoids and Anthocyanins

Using the knowledge obtained, it is interesting to put forth the hypothesis that anthocyanins and iridoids consumed concomitantly may exert positive additive or synergistic effects on dyslipidemia and atherosclerosis. Thus far, only several fruits containing both anthocyanins and iridoids are known. They are, mainly, the cornelian cherry (*Cornus mas* L.), *Cornus officinalis* Sieb. et Zucc., and other *Cornaceae*, honeysuckle berry (*Lonicera caerulea* L.), lingonberry (*Vaccinum vitis-idaea* L.), blueberry (*Vaccinium corymbosum* L.), and cranberry (*Vaccinium oxycoccos* L.). Researchers found that oral administration of lyophilisated cornelian cherry fruits (with substantial amounts of anthocyanins and iridoids, mainly loganic acid) in feed-induced atherosclerotic rabbits prevented the development of dyslipidemia and atherosclerosis through the activation of PPAR-alpha receptors in the liver. Anti-inflammatory properties of whole fruits, measured as a decrease in proinflammatory cytokines were also confirmed [49]. Then, they found that oral administration of cornelian cherry may modulate vascular NO balance by increasing the L-arginine and L-arginine/asymmetric dimethylarginine (ADMA) ratio and decreasing endogenous inhibitors of endothelial nitric oxide synthase- (eNOS-) ADMA and symmetric dimethylarginine (SDMA). These effects were accompanied by a decrease in intima thickness and the intima/media ratio and an increase in GSH level in the thoracic aorta [47]. In a subsequent study done with the same animal model, Sozański et al. demonstrated that both loganic acid and anthocyanins isolated from cornelian cherry fruits diminished dyslipidemia, increased the expression of PPAR-alpha and PPAR-gamma receptors (Figure 2), and decreased intima thickness and the intima/media ratio in the thoracic aorta. Although the degree of these effects differed between anthocyanins and iridoids, the trend in changes was similar. The only significant difference between anthocyanins and iridoids was that it was mainly loganic acid exerting anti-inflammatory effects [48]. Further analyses are required to indicate which iridoids and anthocyanins and at what doses have the most beneficial anti-inflammatory effect.

## 5. Future Perspectives

Incorrect eating habits can be called a peculiar epidemic of the 21st century. Fortunately, thanks to the louder calls for change and the slow but steadily increasing public awareness of healthy nutrition, the share of plant products in the diet of many people is increasing. Thanks to this, compounds with proven therapeutic effects on the body, such as iridoids or anthocyanins, may play an increasingly important role in the prevention and adjunctive or combined treatment of many civilization diseases. The undoubted advantages of using iridoids and anthocyanins in medicine include the fact that many of their effects and mechanisms of action are relatively well known and have been confirmed by many studies. Unfortunately, those were largely animal studies and certainly one of the most important goals for the near future is to conduct more clinical studies to confirm these effects in humans. New applications for the abovementioned compounds should also be sought, as they are potentially a valuable option for usage in, e.g., metabolic syndrome. However, it should be remembered that as natural products, iridoids and anthocyanins will prove best in the prevention of early stages of liver and cardiovascular diseases. Moreover, their complete effect will depend on properly selected doses with confirmed pharmacological effects of a single compound or a mixture of these compounds, the length of therapy, and the regularity of use by patients.

## 6. Conclusion

In conclusion, current knowledge clearly points to the benefits of consuming foods comprising both iridoids and anthocyanins. These compounds exert many curative ascendancies, including pleiotropic positive effects, modulation of transcription factors regulating lipid and glucose metabolism, redox stress and inflammation, and impact on NO vascular balance. The abovementioned findings justify future studies that are aimed at better understanding the properties of currently known iridoids and anthocyanins or creating new phytopharmaceuticals containing both groups of substances.
